# Evaluating the Performance of 3D-Printed PLA Reinforced with Date Pit Particles for Its Suitability as an Acetabular Liner in Artificial Hip Joints

**DOI:** 10.3390/polym14163321

**Published:** 2022-08-15

**Authors:** Ahmed Fouly, Ibrahim A. Alnaser, Abdulaziz K. Assaifan, Hany S. Abdo

**Affiliations:** 1 Department of Production Engineering and Mechanical Design, Faculty of Engineering, Minia University, Minya 61519, Egypt; 2 Mechanical Engineering Department, College of Engineering, King Saud University, Riyadh 11421, Saudi Arabia; 3 King Abdullah Institute for Nanotechnology, King Saud University, P.O. Box 2455, Riyadh 11451, Saudi Arabia; 4 Center of Excellence for Research in Engineering Materials (CEREM), King Saud University, P.O. Box 800, Riyadh 11421, Saudi Arabia; 5 Mechanical Design and Materials Department, Faculty of Energy Engineering, Aswan University, Aswan 81521, Egypt

**Keywords:** PLA composite, hip joint replacement, date pit filler, 3D printing, fused filament fabrication

## Abstract

Off-the-shelf hip joints are considered essential parts in rehabilitation medicine that can help the disabled. However, the failure of the materials used in such joints can cause individual discomfort. In support of the various motor conditions of the influenced individuals, the aim of the current research is to develop a new composite that can be used as an acetabular liner inside the hip joint. Polylactic acid (PLA) can provide the advantage of design flexibility owing to its well-known applicability as a 3D printed material. However, using PLA as an acetabular liner is subject to limitations concerning mechanical properties. We developed a complete production process of a natural filler, i.e., date pits. Then, the PLA and date pit particles were extruded for homogenous mixing, producing a composite filament that can be used in 3D printing. Date pit particles with loading fractions of 0, 2, 4, 6, 8, and 10 wt.% are dispersed in the PLA. The thermal, physical, and mechanical properties of the PLA–date pit composites were estimated experimentally. The incorporation of date pit particles into PLA enhanced the compressive strength and stiffness but resulted in a reduction in the elongation and toughness. A finite element model (FEM) for hip joints was constructed, and the contact stresses on the surface of the acetabular liner were evaluated. The FEM results showed an enhancement in the composite load carrying capacity, in agreement with the experimental results.

## 1. Introduction

Although human age is the main reason for osteoarthritis, being overweight accidents can increase osteoarthritis risk [1,2]. Osteoarthritis of hip joints dramatically impacts human health and can result in affliction and disability, sometimes requiring high-cost treatment [3]. With respect to enhancing quality of life of those who suffer from end-stage osteoarthritis of the hip joints, total replacement of the hip joint is considered the most effective treatment with a reasonable cost; it is anticipated that hip joint replacement will remain the leading treatment choice in the future [4]. According to a statistical study conducted in the United Kingdom, 83,886 osteoarthritis patients underwent a total hip replacement surgery in 2015 [5]. Another study showed that more than 300,000 osteoarthritis patients undergo hip operations in the United States annually [6]. Consequently, developing a highly reliable and durable hip joint prosthesis is essential to decrease the impact on hip joint replacement patients. Furthermore, such a reliable hip joint could enhance a patient’s quality of life and reduce the need for postoperative maintenance or a revisionary operations. To investigate the design of such joint prostheses, it is necessary to understand the nature of the material used for their manufacture. Researchers, engineers, and companies have sought to develop implants/joints with sufficient characteristics. However, the utilized materials still have low durability and relatively limited reliability because of insufficient mechanical properties, resulting in postoperative instability.

Ceramic materials, such as alumina, zirconia, and silicon carbide, have been utilized to fabricate hip joints, owing to their desirable tribological properties [7,8]. However, the brittleness of ceramics represents the main barrier to their in use in joint protheses. Researchers reported that 2% of joint fractures occur due to the brittleness of ceramics [9]. Another study revealed that more than 20% of hip joint patients suffered from squeaking artificial ceramic prostheses [10]. Metallic joint implants are the second choice in joint design due to their good mechanical properties and design flexibility [11]. Researchers found that titanium and tantalum metals, as well as their alloys, can spontaneously bind to bones after special treatment. Such an advantage encouraged researchers to use such metals and their alloys to fabricate artificial joints; such materials are called bioactive metals [12]. However, joint degradation due to the synergy between corrosion and wear on joint surfaces limits the reliability and durability of the joint. Furthermore, the interaction between wear debris and human tissue could result in adverse complications. Multiple investigations have revealed that metal debris other than metal ion leaching led to considerable inflammation and necrosis in the tissue surrounding the artificial joint, which could increase patients suffering [11,13,14,15]. In April 2010, medical regulatory agencies in the United Kingdom issued alerts with respect to the utilization of metal-on-metal hip joints [16]. In February 2011, United States medical agencies announced that the problem with the utilization of metal-on-metal implants was associated with specific designs, and many implants have worked effectively with patients for a long time [17].

Currently, polymers are used in a wide range of applications as versatile structures with many merits [18]. Furthermore, polymers can are applied in various fields, such as sensors [19], biophysics, electronics, medicine, and other branches of engineering science. These polymers can be used in biomedical applications as biopolymers, biomedical polymers, and smart polymers [20]. Such biomedical polymers are characterized by unique properties, such as long shelf life, ease of processing, lightweight, sterilization ability, and reasonable mechanical characteristics, making them suited for specific applications. In 1949, Sir Nicholas Harold, a British ophthalmologist, fabricated an intraocular lens from poly(methyl methacrylate), which was the first reported use of a polymer in medical applications [21]. Two parameters were selected to qualify any material for biomedical use: biodegradability and biostability. According to these criteria, many polymers, such as polyanhydrides, poly(amido amines), polyesters, poly(β-amino esters), poly(ortho esters), and polyamides, were categorized as biomedical polymers due to their hydrolytic degradability [22]. Because of the wide-ranging use of polymers in various biomedical applications, there is no ideal choice or polymer family that can be specified as a biomedical polymer. However, researchers established a library of polymeric materials that are available and can be synthesized according to the biomedical application and desired function [22].

Regarding the utilization of polymers in artificial hip joints, polymer-on-metal approaches are conventionally used, whereby an acetabular liner of ultrahigh molecular weight polyethylene (UHMWPE) moves relative to a femoral head made of a cobalt-chromium-molybdenum (Co-Cr-Mo) alloy. However, the mechanical properties of UHMWPE are poor, encouraging the erosion of the acetabular liner. Moreover, the wear contaminants can lead to a host of inflammation, triggering osteolysis [23,24]. Furthermore, the complexity of the production process limits the design features, and such a problem can lead to a mismatching on the surfaces of the joint elements, and creep under cyclic load can rupture the UHMWPE part [25]. Kim et al. [26] reported that in the United States, during total hip replacement surgery, the surgeon removes the deteriorated implant and places an antibiotic PMMA-eluting spacer block. Then, they reimplant a new implant, which is treated with intravenous antibiotics. However, they found that the mechanical properties of PMMA as a spacer biopolymeric material are insufficient for such applications due to its brittleness [27]. Furthermore, the PMMA polymerization reaction is substantially exothermic; consequently, it limits the utilization of heat-stable antibiotics [28]. Thus, it is essential to explore new materials with acceptable mechanical properties, as well as ease of fabrication, which allows for flexibility in the process.

To overcome the execution of design complexity, additive manufacturing approaches (3D printing) can be used. Additive manufacturing represents a revolution in engineering design, enabling several interesting approaches to implement customized biomedical parts, including the development of human organs [29], femoral implant rods [30], oral and maxillofacial surgery [31], and electronic ears [32]. Polylactic acid (PLA) is considered one of the most famous materials in 3D printing technology [33]. Besides minimal warping problems, the ease of utilization makes PLA an optimal choice for 3D printing. Polylactic acid (PLA) is a bio-based polymer that can be extracted by fermenting agricultural materials, such as potato, corn, and sugar beet [34]. Consequently, PLA is environmentally friendly, and researchers consider it a biomedical polymer [35]. PLA has a good barrier ability, can be easily processed, and possesses acceptable mechanical properties. However, PLA still requires some modifications to enable its use in commercial applications, such as modifications to its low-impact strength, stiffness, and strength [36].

To enhance their mechanical, thermal, and physical properties, researchers have sought to add fillers or fibers to polymers [37,38]. Huda et al. [39] illustrated that the addition of fillers to PLA can affect all the properties of the produced composite. Researchers found that incorporating ceramic, wood, carbon, and metals in 3D-printed PLA can enhance the mechanical properties of the produced composite in with respect to material deposition [40]. However, using natural fibers and/or fillers is recommended to produce a PLA composite with properties suitable for biomedical applications [41]. 

Natural additives have attracted the attention of researchers due to their ease of availability in nature with approximately no cost, ease of manufacturing, biocompatibility, and eco-friendliness. Liu et al. [42] reinforced PLA with eucalyptus, newspaper, pine, lignin, and pulp, with various loading fractions, from 0 to 20%, and investigated the effect on its mechanical properties, quality of printing, and melt flow index. Another study evaluated the mechanical and rheological properties of 3D-printed PLA after reinforcing it with beechwood at loading fractions of 0 to 50% [43]. Tisserat et al. [44] investigated the effect of reinforcing PLA with Osage orange wood and Paulownia wood. They reported a slight enhancement in Young’s modulus and elongation. However, a deterioration in the tensile strength occurred compared with pure PLA.

Date palm is considered a principal fruit and is planted in various regions around the world. Furthermore, dates are considered a strategic crop in some countries, such as Saudi Arabia. However, using large amounts of dates in such countries results in overflowing by products of date pits [45]. Date pits represent approximately 10–25% of date fruit composition [46]. In some countries, such as the United States, date pits are considered a waste by product of the date industry; therefore, solutions are required with respect to their disposal [45]. Recently, researchers attempted to use date pit waste as a natural filler for the development of composites. Preliminary investigations of date pits as filler to produce material composites showed that, compared with some lignocellulosic residues, such as pistachio flax fiber and shell flour, date pits as fillers have a superior melt flow index [47]. Basim et al. [48] reported the conversion of date palm seeds into a filler and used it as a reinforcement for unsaturated polyester. They incorporate up to 70% the date seeds into a polyester matrix before applying a thermoset curing process. They found that the incorporation of up to 50% date seeds resulted in low thermal conductivity and diffusivity. However, mechanically, both compressive and tensile strength were enhanced. Sismanoglu et al. [49] developed an eco-grade polyurethane composite by adding 46% date palm seed. They used alkali and silane to modify the surface of the date seed powder to improve the compatibility between the polyurethane and date seed powder. They reported an enhancement in the mechanical properties of the composite, in addition to an improvement in the storage modulus and thermal stability. Furthermore, date pits have piqued the attention of many researchers due to their biodegradability and distinguished biocompatibility [50,51].

We believe that there is a need to develop a new composite that can be used for hip joints with satisfactory mechanical properties to allow for flexibility in the design process and accuracy in the fabrication process. Therefore, we selected polylactic acid (PLA) due popularity for the 3D printing techniques, allowing for design flexibility and high precision in production. Given the formidable merits and opportunities associated with natural materials, further investigation is required to evaluate the mechanical behavior of natural-material-based PLA composites. Hence, we selected date pits/seeds as the raw material for the current study. To the best of our knowledge, no standard data are available regarding the conversion of date pits/seeds from primary form as natural waste into a specific usable filler that can be used in polymer composites. Furthermore, no data are available concerning their use without any purification treatment, which could be costly, eliminating the valorization concept. Consequently, we designed a complete production process for date pit particles. The produced date pit powder properties were estimated using XRD and SEM. The mechanical properties of PLA incorporated with date pit particles at various loading fractions were evaluated and in terms of hardness, compressive ultimate strength, modulus of elasticity, elongation, and modulus of toughness. A finite element model for hip joints including an acetabular liner with the mechanical properties extracted from the experimental work was built to evaluate the generated stress of the new composite element under the weight of an actual human.

## 2. Materials and Experimental Work

### 2.1. Materials

PLA, a 100% new raw material with minimal warping, no impurity, and minimal shrinkage mainly made of corn starch, was supplied by Shanghai Nuolei CNC Router Equipment Co., Ltd., Shanghai, China. Consequently, it is considered to be environmentally an user-safe (eco-friendly). It comes in the shape of a filament with a diameter of 1.75 mm with a density of 1.25 g/cm^3^ and can be printed at temperatures ranging from 190 °C to 220 °C. Date pits/seeds were supplied by Al Khmash Company for pressed dates, Al Qassim farms, Saudi Arabia. There are many types of date fruit, and the properties of the date pits may differ according to the date fruit type. In the current study, the utilized date pits were extracted from a popular date fruit called the Kholas date. The utilized seeds usually consist of 8.6–12.5% moisture, 5.7–8.8% lipids, 4.8–6.9% protein, 0.8–1.1% ash, 67.6–74.2% dietary fiber, and 2.4–4.7% carbohydrates [52].

### 2.2. Preparation of Date Pit/Seed Powder

**Figure 1:**
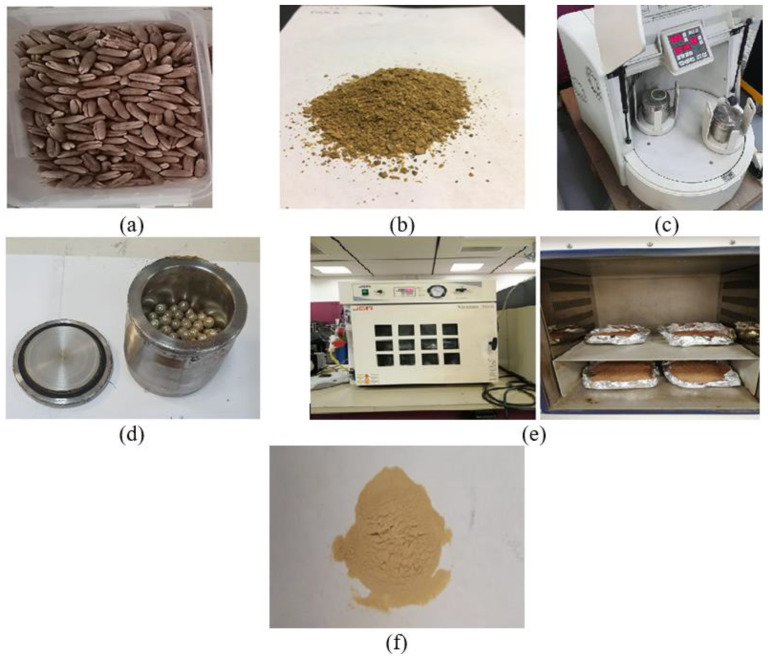
Preparation of date pit particles: (**a**) raw date pits after initial drying, (**b**) date pit particles after initial grinding process, (**c**) ball milling machine, (**d**) balls and date pit powder inside the ball milling jar, (**e**) final drying process, and (**f**) date pit power as a filler.

Date pits were initially washed with water and detergent (Aston) to remove any sticky contaminants and date molasses. Then, the date pits were left to dry in the summer sun for one month before drying inside a vacuum oven (JSV0-60T) at 60 °C for one week to remove any moisture. Then, the date pits were ground into small parts using a mortar and pestle. The small pieces were pulverized with a grain miller at 200 rpm, and the produced powder was transferred to the jar of a ball milling machine (Pulverisette 7, Fritsch, Idar-Oberstein, Germany). A stainless-steel ball was utilized to mill the date pit particles to a fine powder. The weight ratio of steel balls to date pit particles was 10:1; steel balls (100 gm) were assumed to mill 10 gm of date pit particles. The rotation speed of the ball milling machine was set to 200 rpm, and the milling time was set to 8 h, with 15 min of continuous milling followed by 15 min of rest to avoid high heat generation inside the milling jar. After finishing the ball milling process, the balls were ejected and cleaned inside an ultrasonic bath to remove any stuck powder on the ball’s surface to be prepared for the next 10 g of date pits. The achieved powder was dried in a vacuum oven for 24 h to ensure complete removal of any moisture. Figure 1 shows the date pit powder production process.

### 2.3. Fabrication of Date-Pit-Reinforced PLA Biopolymer

**Figure 2:**
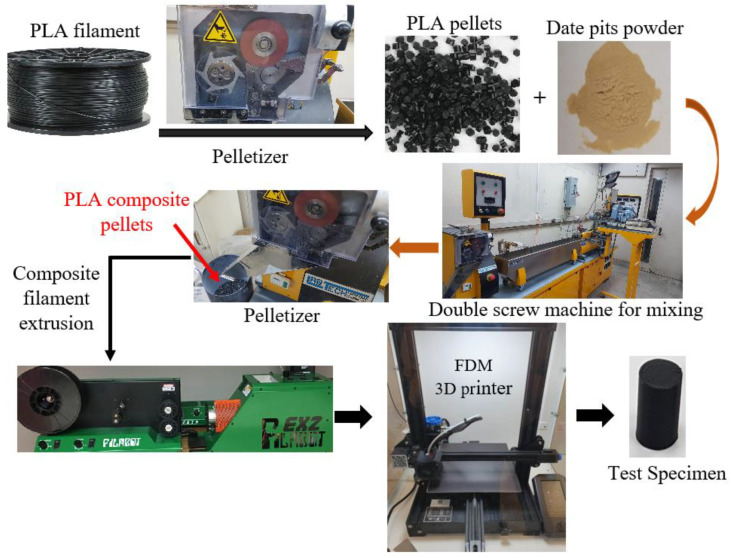
Flowchart of PLA–date pit composite filament production and the fabrication of test specimens using an FDM 3D printer.

PLA filament was first fed into a pelletizer to produce PLA pellets. Then, the PLA pellets were weighted and mixed with date pit powder at varying weight fractions, from 0 to 10 wt.%, in 2% increments. The two materials were first mixed utilizing a mechanical stirrer at a speed of 300 rpm for 20 min to disperse the date pit powder into the PLA matrix at room temperature. Then, the mixture was fed into a twin extruder and extruded through ten temperature zones ranging from 160 °C to 210 °C at a speed of 50 rpm. The diameter of the screw of the extruder was 16 mm, and the length-to-diameter ratio was 40 L/D. Once the mixture passed through the capillary die, the extruded blend was cooled in a water bath before passing through the pelletizer again. The mixed pellets were n and fed through the twin extruder again to ensure homogenous dispersion of the date pits inside the powder. The previous process was repeated three times for each composition. No additional binding assistive material was during the composite production process. The final PLA composite pellets were fed into a Filabot EX2 single-screw extruder to fabricate PLA–date pit composite filaments suitable for FDM 3D printing. Finally, test specimens were fabricated using a 3D printer (Creality 3D^®^ Ender-3 V2, Shenzhen creality 3D Technology Co., Ltd., Shenzhen, China). Figure 2 shows a flowchart of PLA–date pit composite filament production and the fabrication of test specimens using an FDM 3D printer. The sample was created using SolidWorks and imported to Cura software to model modification and G-code generation. The 3D printer settings were tuned to produce specimens with 100% density; the nozzle temperature ranged between 200 °C and 210 °C, whereas the bed temperature was set to 70 °C. Although 3D printing parameters have a significant effect on the properties of the 3D printed specimens, we held all parameters equal for all specimens, with the goal of only investigating the effect of reinforcing PLA with date pit particles on the mechanical properties of the 3D-printed composite samples. The PLA composite samples with date pits weight fractions of 0, 2, 4, 6, 8, and 10 wt.% are referred to as PLA-DP0, PLA-DP2, PLA-DP4, PLA-DP6, PLA-DP8, and PLA-DP10, respectively.

### 2.4. Characterization and Testing

The crystallinity of the produced date pit powder was evaluated using an X-ray diffractometer (XRD) with D8 discover from Bruker, Germany. The morphology of the produced powder was observed using field-emission scanning electron microscopy (model: JEOL JSM-7600F, Tokyo, Japan). The crystallinity of the produced PLA-DP composites was evaluated using an X-ray diffractometer (XRD). Furthermore, thermal decomposition patterns of the PLA-DP composites were obtained using thermogravimetric TGA on a TA-Q500 System of TA. Approximately 10 mg of each PLA-DP composite sample was heated with a heating rate of 10 °C/min from 30 to 800 °C under a nitrogen atmosphere utilizing TG-DTA (NETZSCH Germany (Model: STA 449 F3)).

The densities of PLA–date pit composites were measured experimentally based on Archimedes’ principle [53]. The weight of PLA–date pit composite samples was recorded in an air atmosphere and after being submerged in alcohol; consequently, the density was calculated according to the following equation:

$$\rho_{PLA-DP}=\left(\rho_{alc}-\rho_{air}\right)\times \frac{m_{air}}{m_{air}-m_{alc}}+\rho_{air}$$

where *ρ_PLA-DP_*, *ρ_alc_*, and *ρ_air_* are the experimentally measured densities (g/cm^3^) of the PLA-DP composites, alcohol, and air, respectively; and *m_air_* and *m_alc_* are the masses of the PLA-DP composites (g) in air and alcohol, respectively. The density of each composite sample was measured 5 times, and the average density was calculated.

The hardness of the prepared samples was measured using a Shore D durometer, which has a capacity of 5 ± 0.5 kg and a response time of 15 s according to ASTM D2240 [54]. The hardness was measured 6 times for each sample on different regions on the surface of the composite samples before calculating the average hardness. Furthermore, the PLA-DP composite samples were prepared according to ISO 604 Plastics [55], with a length of 16 mm and a diameter of 8 mm to be compressed and extract the compressive mechanical properties. An Instron 5582 microtester (Instron, University Ave, Norwood, MA, USA) was used for the compression test, and the deformation rate was tuned to 2 mm/min. After the test, the compressive mechanical properties were extracted from the stress–strain diagram, the modulus of elasticity, compressive ultimate strength, modulus of toughness, and the elongation. A compression test was performed 5 times for 5 samples of each composite, the average values were recorded from the experimental results, and the standard errors were calculated for all mechanical properties.

### 2.5. Hip Joint Finite Element Model

**Figure 3:**
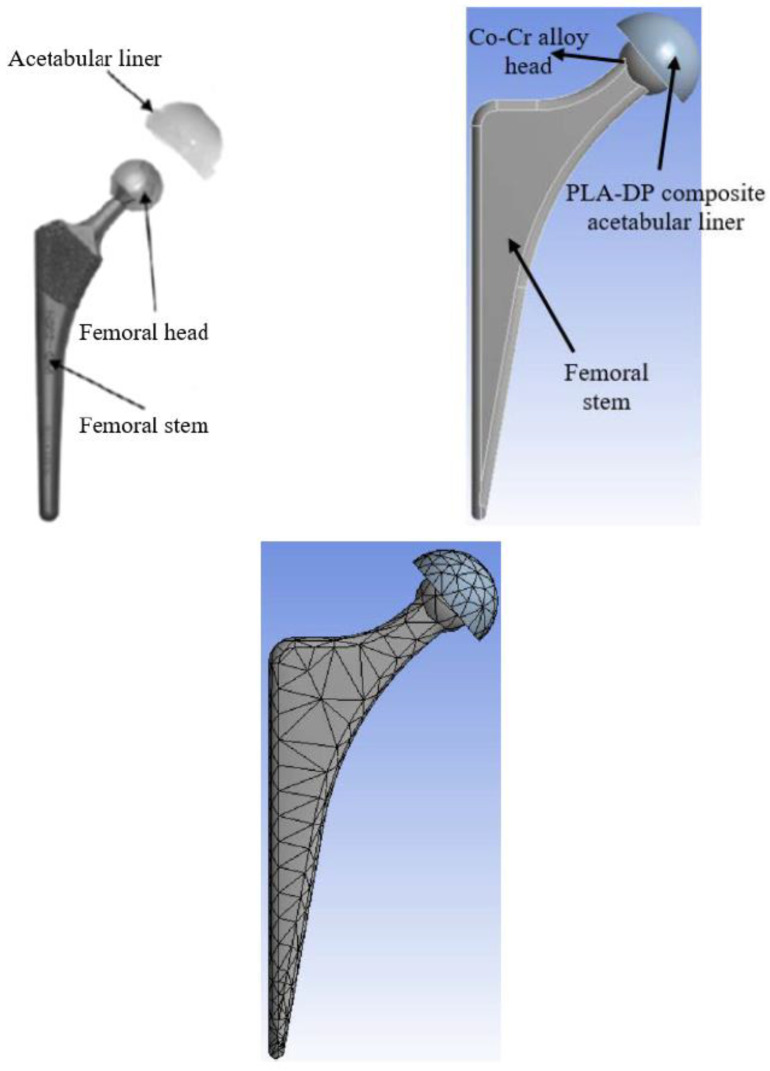
Schematic of the artificial hip joint and the finite element model of the hip joint with and without meshing.

In order to investigate the load-carrying capacity of the new PLA-DP composites when used as an acetabular liner in an artificial hip joint, a finite element model was constructed. According to a study conducted in Japan by Ishihara [56], an artificial hip joint could be modeled in two main parts: femoral head made of Co-Cr alloy or ceramic ball and a polymeric acetabular liner, as shown in Figure 3. Consequently, using ANSYS software, a hip joint was constructed, as shown in Figure 3. A ball was blended to the femoral stem, and an acetabular liner was made of the proposed PLA-DP composite. A combination of tetrahedron and hexahedron shapes was used to mesh the whole model (automatic mesh). The entire model consists of 1247 elements and 2525 nodes. The solver used in the current study is a mechanical APDL, the hip joint is modeled in 3D, and a SOLID 185-element type is defined for the whole structure. Furthermore, CONTACT173 and TARGET170 elements were used as elements used to define the contact between the acetabular liner and the femoral head. In order to define the boundary conditions, the loading conditions were extracted from a study conducted by Bergmann et al. [57], which showed that there is a compound load on the surface of the hip joints, defined in x, y, and z directions for various types of motion. For a human body weight of 100 Kg in a standing position, the maximum stumbling resultant forces are 1540, 3480, and 70 N in the x, y, and z directions, respectively. On the other hand, the femoral stem with the Co-Cr ball was fixed in all directions. The contact between the ceramic ball and the PLA-DP composite acetabular liner was defined as bonded. The mechanical characteristics of the PLA-DP composite acetabular liner were defined based on the experimental results.

## 3. Results and Discussion

**Figure 4:**
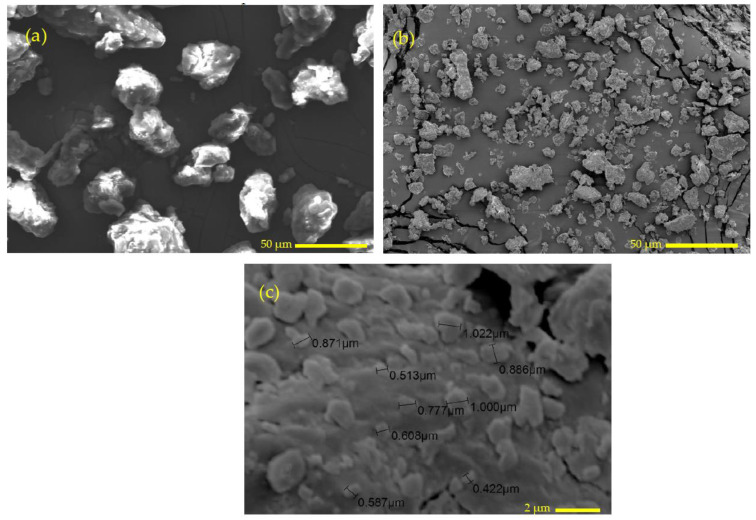
SEM image of date pit particles (**a**) after the grain milling process and (**b,c**) after the ball milling process.

As mentioned with respect to the preparation of the date pit powder, the grinding of the date pit powder involved two stages. Figure 4a is a SEM image of the morphology of date pit particles after being processed by the grain miller. It is obvious that the date pit particles have irregular equiaxed shapes with rough of particle surfaces. The particle size varies between 25 µm and 50 µm. Figure 4b shows the morphology of the date pit particles after the ball milling process. The date pit particles became smaller and flattened, which could be attributed to the compound forces to which the date pit particles were subjected, such as the compressive and shear forces due to the movement of the balls inside the jar. Such compound forces can break the date pit particles and flatten the resultant small particles. Figure 4c illustrates the size of the date particles after the ball milling process, with an average size of 0.6 µm and a flaky shape.

**Figure 5:**
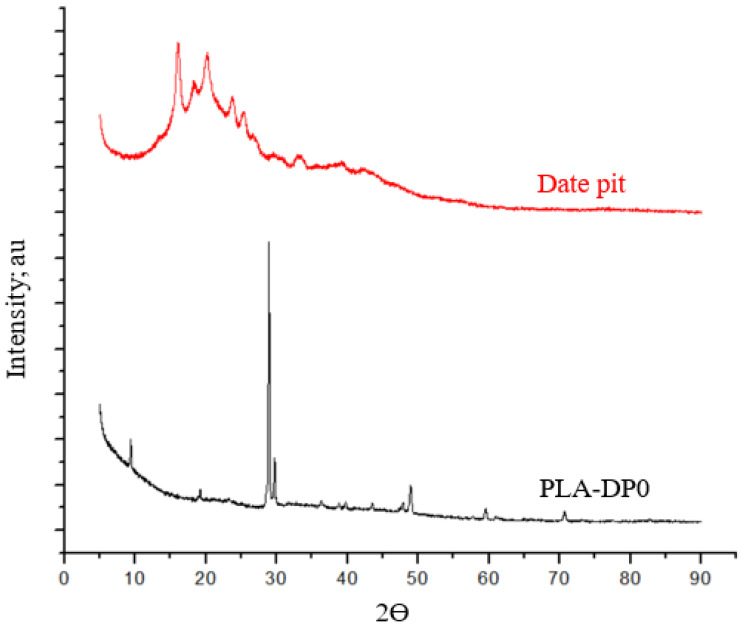
X-ray diffraction (XRD) traces of pure PLA and date pit powder.

**Figure 6:**
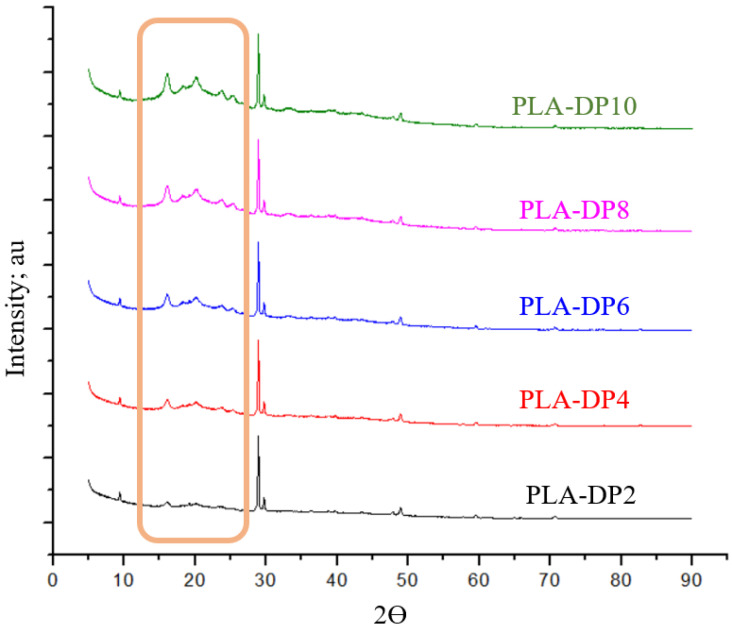
X-ray diffraction (XRD) traces of PLA–date pit composites.

To analyze the effect of date pit incorporation within the PLA polymer on the chemical composition of the composite, X-ray diffraction (XRD) was applied for pure PLA, date pit powder, and each produced composite, as shown in Figure 5 and Figure 6. The XRD of the utilized PLA in the current research demonstrates several peaks at 2θ angles of 9.5° and 28.5°, similar to the results reported by Kumar et al. [58]. The appearance of a peak at 9.5° for the PLA indicates that the supplier had added cereal starch to increase the biodegradability of the PLA [58]. Utilizing native starch granules was proven to lead to the appearance of two primary types of XRD patterns: one for cereal starches and the other for tuber- and amylose-rich starches [59]. The exhibition of peaks at 9.5° and 28.5° indicates the existence of cereal starch extracted from potato, wheat, and tapioca. Otherwise, the diffractogram of date pits illustrates that amorphous nature is the main property of the matter. The appearance of some diffraction peaks indicates a minimal amount of crystalline matter. The date pit XRD is in line with the results reported by Abed et al. [60]. The incorporation of date pits into the PLA matrix affected the general contour of the composite, illustrated by a combination of PLA peaks and date pit peaks, as shown in Figure 6. All XRD patterns of the PLA-DP composites demonstrate similar crystalline peaks, although the intensity of date pits peaks varied depending on the date pit concentration, as shown in the orange rectangle, emphasizing the thorough distribution of date pit powder inside the PLA. The lack of new peaks implies that no chemical reaction occurred between the matrix and filler [61] and that the presence of date pit powder did not affect the structural properties of the PLA.

**Figure 7:**
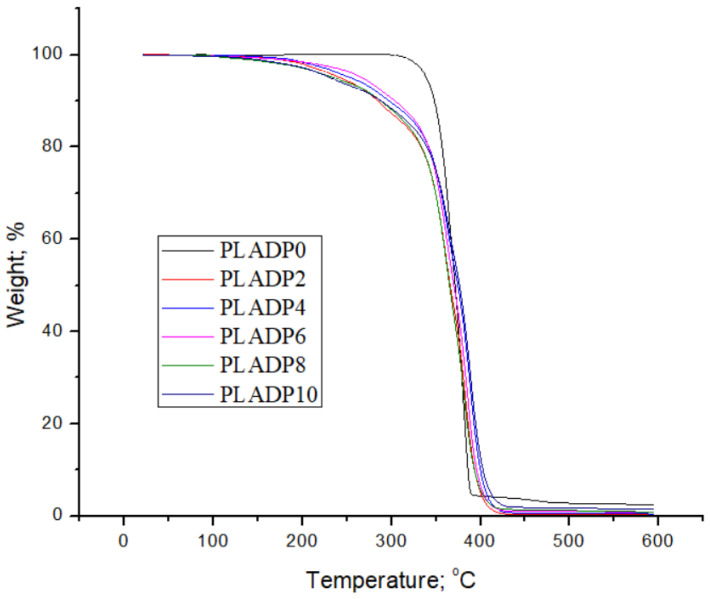
Thermogravimetric curves of pure PLA and PLA–date pit composites.

Figure 7 illustrates the thermal stability of the pure PLA until complete thermal degradation started at approximately 350 °C. On the other hand, the performance of the PLA-DP composite was divided into two regions; the first region of thermal degradation started at 150 °C with a gradual decrease until 335 °C, when a complete thermal degradation occurred. Bajpai et al. [36] illustrated that the weight loss in the first region could be attributed to moisture evaporation, whereas the complete thermal degradation indicates the degradation of the polymer. Following the thermal degradation process of all PLA-DP composites, the remaining weight of the composite formed a shoulder that was approximately fixed after 400 °C. According to some investigations, char is considered the primary component of the residual mass at the end of the thermal degradation process [62,63]. According to the current results, the degradation temperature of all PLA-DP composites is lower than that of pure PLA. The difference in the thermal degradation among the tested composites could be attributed to the nature of the date pits, their surface properties, and the interfacial condition with PLA.

**Figure 8:**
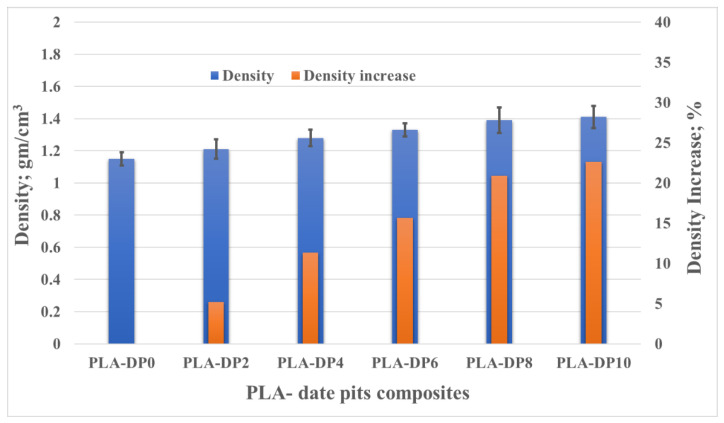
PLA composite density for different date pit contents.

**Figure 9:**
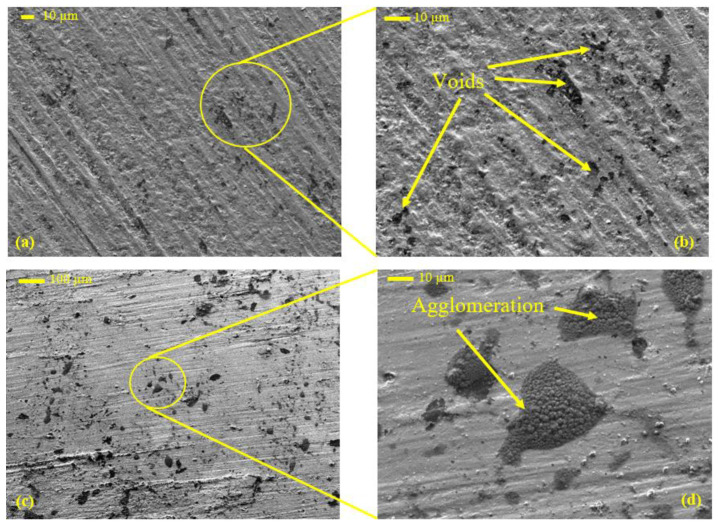
SEM images of the surfaces of (**a**,**b**) pure PLA and (**c**,**d**) PLA-DP10.

Density plays a significant role in evaluating the physical properties of the material. Usually, the density of composites depends on both the matrix and the filler densities. Consequently, the density of the PLA with varying weight fractions of date pits was measured and compared. Figure 8 illustrates the change in the composite density with respect to the change in date pit weight fraction. The density increased linearly with the increased date pit weight fraction, indicating that the density of the date pit powder was higher than that of the PLA. The increase in the PLA composite density occurred due to the replacement of the weight of the PLA with the weight of high-density date pit powder. It has been proven that even if the infill density is set to 100% during 3D printing, there is no guarantee that the samples will have no voids [64]. It seems that the pure PLA specimen had some voids that formed during the fabrication process, as shown in Figure 9a,b. In the PLA composites, the date pit particles may have agglomerated and filled the voids of the PLA, as shown on the surface of PLA-DP10 in Figure 9b,c. This could explain the dramatic increase in the composite density, which reached 23%.

**Figure 10:**
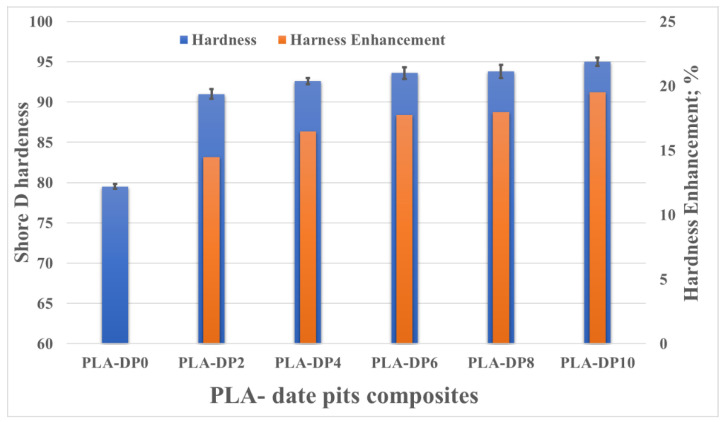
PLA composite harness for different date pit contents.

The shore D hardness of the PLA composite samples with varying weight fractions of date pits is illustrated in Figure 10. The hardness of the composites increased with increased date pit weight fraction, reaching approximately 20% for PLA-DP10 compared with pure PLA. The composite hardness is affected by the intermolecular bonds between the PLA matrix and the date pit powder; improved composite hardness indicates the uniform distribution of the filler particles inside the polymeric matrix [65]. Such uniform distribution enhances the load transfer between the matrix and the filler. The enhanced hardness could also be attributed to the increase in the stiffness with increased date pit weight fraction. Furthermore, incorporating date pit powder in the PLA decreases voids on the sample surface, as shown in Figure 9, representing a resistance against the tip of the durometer during hardness measurement. Finally, Guo et al. [66] reported a relationship between hardness and density whereby increasing density can lead to an increase in hardness.

**Figure 11:**
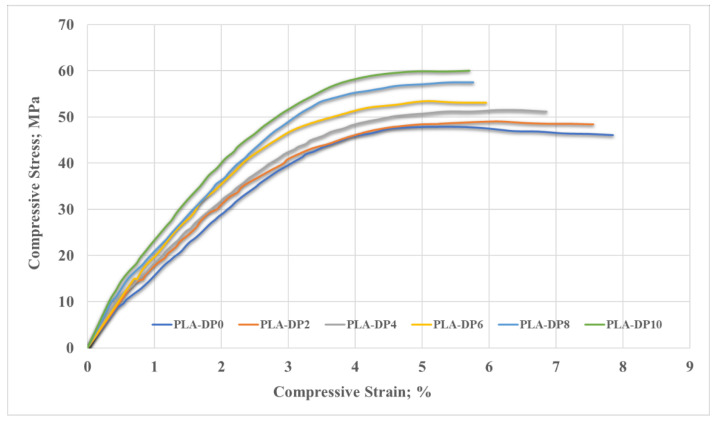
Compressive stress–strain curves of PLA–date pit composites.

**Figure 12:**
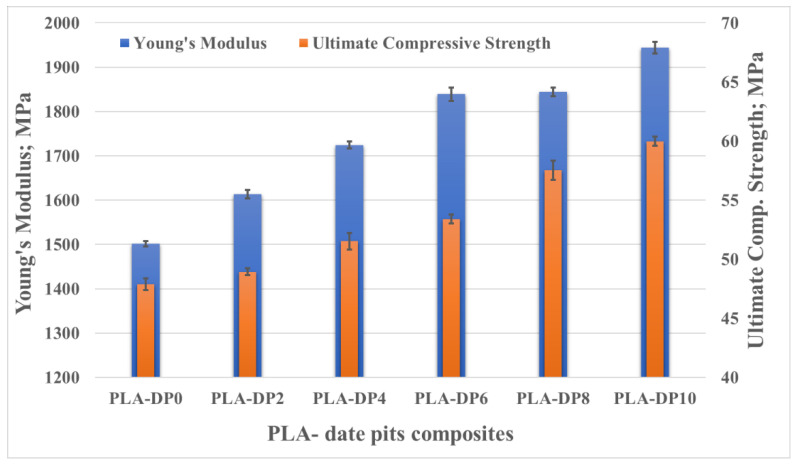
Young’s modulus and ultimate compressive strength of PLA–date pit composites.

Figure 11 shows the stress–strain curves recorded during the compression test of all PLA–date pit composite samples. Incorporating date pit particles in the PLA reduced the flexibility of the PLA chains and improved the PLA compressive properties. The compressive Young’s modulus was extracted for each sample from the linear region of the curves. The ultimate compressive strength was recorded based on the highest measured stress during the test, as shown in Figure 12. PLA-DP10 had the highest compressive Young’s modulus and ultimate strength, at 1944 MPa and 59 MPa, respectively. The enhancement in Young’s modulus and ultimate strength for PLA-DP10 compared with pure PLA reached 22% and 25%, respectively. Additionally, we observed an enhancement in the composite stiffness and a strength increase that were linearly related to the increase in the date pit weight fraction. 

**Figure 13:**
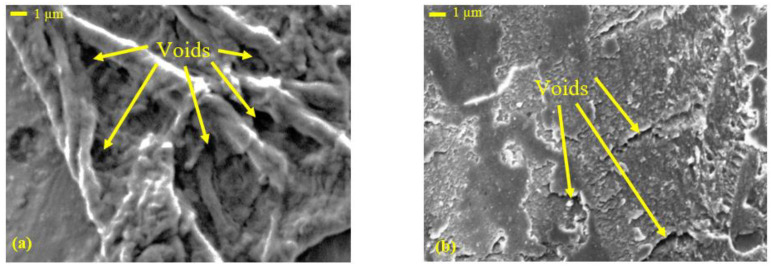
SEM micrographs of the compressive fractured surface of (**a**) PLA and (**b**) PLA-DP10.

The enhancement in the ultimate compressive strength could be attributed to the uniform distribution of the date pits inside the PLA matrix, which encouraged load absorption and dissipation. Furthermore, during the compression test in polymers, as the cracks in the matrix initiated and propagated, the presence of the filler particles healed them and stopped propagation [67]. However, such an increase in strength could affect the material ductility. A comparison of the fracture surface of PLA-DP10 with that of pure PLA showed that the fracture surfaces have different fractographic features, as shown in Figure 13. The morphology of the fracture cross-sectional surface of the pure PLA shows significant voids. Such voids can result in a porous structure, decreasing mechanical properties. The morphology of the PLA-DP10 fractured surface did not show such voids; however, it showed a rough surface. Increasing surface roughness indicates a distortion of a crack path due to the presence of the date pit particles, which illustrates the enhancement in the strength of the composites.

**Figure 14:**
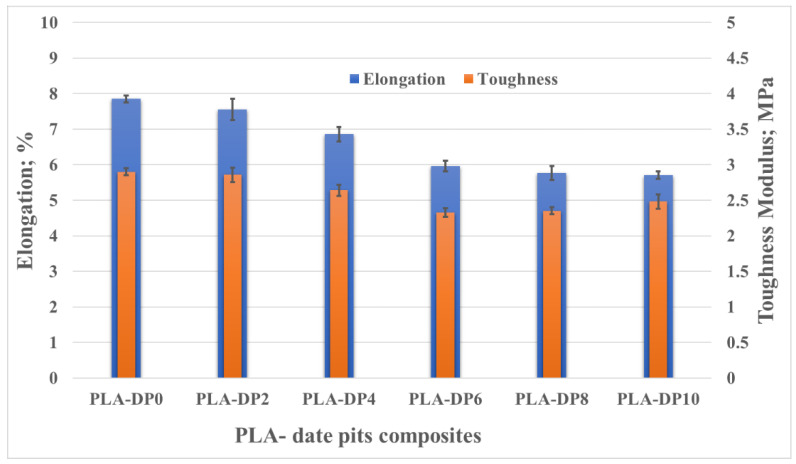
Elongation and toughness modulus of PLA–date pit composites.

As shown in Figure 11, the features of the stress–strain curves indicate the brittleness of all composites, accounting for the low ductility and toughness. Figure 14 illustrates the changes in elongation and toughness modulus with the change in date pit weight fraction. The toughness modulus was calculated based on the area under the stress–strain curve of each composite. However, the elongation was calculated based on the fracture strain. The elongation and toughness decreased with increased weight fraction of date pit particles. The deterioration in the elongation and toughness modulus for PLA-DP10 compared with pure PLA reached 27% and 14%, respectively. The highest elongation was recorded for pure PLA, which could be attributed to the voids inside the pure PLA matrix, which allowed for more deformation. On the other hand, the incorporation of date pit carried some load with the matrix, which enhanced the composite’s strength but prevented the specimen from elongating. Regarding the toughness, the decrease in toughness could be attributed to the low interfacial adhesion between the matrix and the filler [68]. As previously mentioned, in the current study, the date pit particles were not chemically treated with any blend before utilization, which may have affected the adhesion between the PLA and date pit particles.

**Figure 15:**
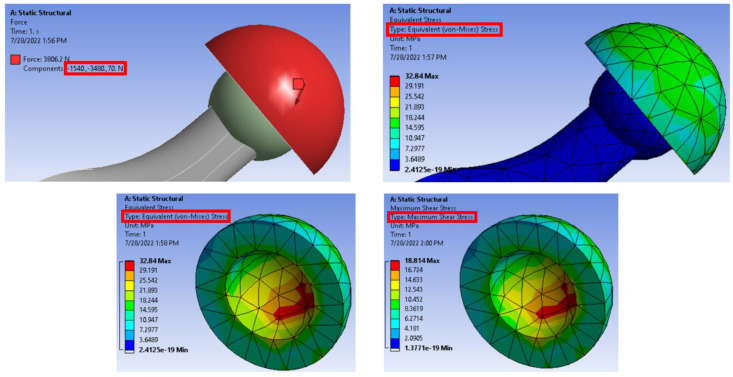
Finite element model of a hip joint with boundary condition, meshing, and contact stresses.

**Figure 16:**
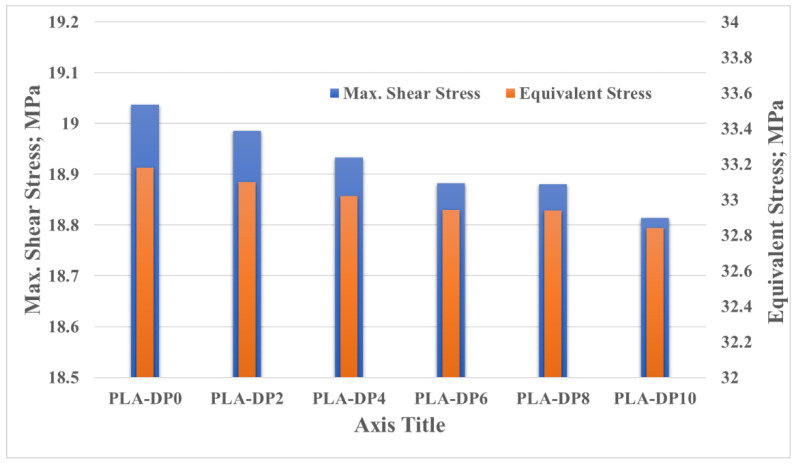
Contact stresses on the surface of the articulated cup of the hip joint.

It is obvious that incorporating date pit particles inside the PLA matrix could enhance some of the mechanical properties. As the composite was developed to be used in an artificial hip joint, the load-carrying capacity of the PLA–date pit composite should be evaluated under actual conditions. Many researchers have evaluated the load-carrying capacity by measuring the contact stresses generated on the surface of the contacted parts [69]. Consequently, an ANSYS model of an artificial hip joint was constructed. The induced stresses due to the weight of a human on the surface of the acetabular liner were estimated, as shown in Figure 15. The boundary conditions were illustrated in Section 2.4 and are indicated in the red rectangle in Figure 15. The generated contact stresses along the outer and inner surfaces of the acetabular liner are presented in the same figure. The highest induced stress appeared on the inside surface, where the acetabular liner is in direct contact with the prosthetic head. The finite element results illustrate that the equivalent von Mises stress and the maximum shear stress varied slightly with the change in date pit weight fraction inside the PLA, resulting in changes in mechanical properties, as shown in Figure 16. Increasing the weight fraction of date pits led to a decrease in both equivalent von Mises stress and maximum shear stress, which reflect the enhancement in the mechanical properties, in line with the experimental results. With lower contact stresses, an enhancement in the load-carrying capacity could be achieved [64]. Furthermore, the maximum generated equivalent stress is 33.18 MPa, approximately half of the experimentally measured ultimate compressive stress; consequently, it is safe to use such newly developed composites in the fabrication of acetabular liners inside artificial hip joints.

## 4. Conclusions

In this investigation, we focused on developing a new polymeric composite that is safe and clean and allows for design flexibility for use as an acetabular liner inside artificial hip joints. A 3D-printed PLA was processed by incorporating natural material, i.e., date pits, within the matrix. We developed a complete production process for the natural filler and composite filament with varying weight fractions of date pits. The various properties of the produced PLA–date pit composites were evaluated. A finite element model representing the artificial hip joint was constructed to evaluate the performance of the acetabular liner with the evaluated composite properties. The results illustrate an enhancement in the hardness, ultimate compressive strength, and Young’s modulus, reaching 20%, 25%, and 22%, respectively, for PLA-DP10 compared with pure PLA. However, the increased date pit weight fraction resulted in a decrease in the elongation and toughness. Furthermore, with increased date pit weight fraction, an increase in the density was noticeable. The finite element results showed that increasing the date pit weight fraction decreased the equivalent von Mises stress and the maximum shear stress generated on the surface of the acetabular liner. The decrease in the contact stresses illustrates an enhancement in the load-carrying capacity, which is in line with the enhancement of the ultimate strength of the composite. In the future, we recommend further analysis of the tribological properties of the PLA–date pit composite to specify the specific wear rate due to friction. 

## Data Availability

Not applicable.
